# Preventive Allergen-Specific Vaccination Against Allergy: Mission Possible?

**DOI:** 10.3389/fimmu.2020.01368

**Published:** 2020-07-07

**Authors:** Inna Tulaeva, Bernhard Kratzer, Raffaela Campana, Mirela Curin, Marianne van Hage, Antonina Karsonova, Ksenja Riabova, Alexander Karaulov, Musa Khaitov, Winfried F. Pickl, Rudolf Valenta

**Affiliations:** ^1^Division of Immunopathology, Department of Pathophysiology and Allergy Research, Center for Pathophysiology, Infectiology and Immunology, Medical University of Vienna, Vienna, Austria; ^2^Laboratory of Immunopathology, Department of Clinical Immunology and Allergology, Sechenov First Moscow State Medical University, Moscow, Russia; ^3^Center for Pathophysiology, Infectiology and Immunology, Institute of Immunology, Medical University of Vienna, Vienna, Austria; ^4^Division of Immunology and Allergy, Department of Medicine Solna, Karolinska Institutet, Karolinska University Hospital, Stockholm, Sweden; ^5^NRC Institute of Immunology FMBA of Russia, Moscow, Russia; ^6^Karl Landsteiner University of Health Sciences, Krems an der Donau, Austria

**Keywords:** vaccine, vaccination, allergy, allergen, allergen-specific immunotherapy, therapeutic vaccine, molecular allergy vaccine, IgE

## Abstract

Vaccines for infectious diseases have improved the life of the human species in a tremendous manner. The principle of vaccination is to establish *de novo* adaptive immune response consisting of antibody and T cell responses against pathogens which should defend the vaccinated person against future challenge with the culprit pathogen. The situation is completely different for immunoglobulin E (IgE)-associated allergy, an immunologically-mediated hypersensitivity which is already characterized by increased IgE antibody levels and T cell responses against *per se* innocuous antigens (i.e., allergens). Thus, allergic patients suffer from a deviated hyper-immunity against allergens leading to inflammation upon allergen contact. Paradoxically, vaccination with allergens, termed allergen-specific immunotherapy (AIT), induces a counter immune response based on the production of high levels of allergen-specific IgG antibodies and alterations of the adaptive cellular response, which reduce allergen-induced symptoms of allergic inflammation. AIT was even shown to prevent the progression of mild to severe forms of allergy. Consequently, AIT can be considered as a form of therapeutic vaccination. In this article we describe a strategy and possible road map for the use of an AIT approach for prophylactic vaccination against allergy which is based on new molecular allergy vaccines. This road map includes the use of AIT for secondary preventive vaccination to stop the progression of clinically silent allergic sensitization toward symptomatic allergy and ultimately the prevention of allergic sensitization by maternal vaccination and/or early primary preventive vaccination of children. Prophylactic allergy vaccination with molecular allergy vaccines may allow halting the allergy epidemics affecting almost 30% of the population as it has been achieved for vaccination against infectious diseases.

## Background

Since the classical experiment in which Edward Jenner vaccinated for the first time with cowpox to protect against smallpox infections in 1796 much has been achieved not only in the field of vaccination against infectious diseases (1–3) but also against cancer (4). With the introduction of vaccines, dramatic drops of episodes of close to or 100% in the cases of infectious diseases were noted demonstrating how effective vaccination can be (4). Vaccination against infectious diseases saves millions of lives every year and has changed living conditions completely and eventually has made a major contribution to increased life-expectancy (3). As far as infectious diseases and cancer are concerned, the purpose of vaccination is to induce a strong adaptive immunity at the antibody and T cell level against pathogens and tumor antigens to protect the vaccinated subject. Everybody would thus assume that vaccination is only useful when it is necessary to create a strong immune response in somebody lacking immunity or at least to enhance the immune response. Accordingly, there are hardly any vaccination strategies available for diseases which are characterized by hyper-immunity such as autoimmune diseases. The main exception is allergen-specific immunotherapy (AIT) for IgE-associated allergies (5). AIT is based on the administration of the disease-causing allergens to induce a “counter immune response” consisting of allergen-specific IgG antibodies, which block binding of IgE to the allergens, and alterations in the cellular immune response, particularly a reduction of allergen-specific Th2 responses (6).

### The Allergic Immune Response

Unlike non-allergic individuals, who mount normal IgG responses upon contact with environmental antigens, allergic patients produce IgE antibodies against allergens (7, 8). Whether an individual develops allergen-specific IgE antibodies or not depends on a large variety of host and environmental factors including genetic factors predisposing for IgE production as well as allergen exposure and adjuvant factors to just name a few (9). IgE antibodies belong to the least abundant class of immunoglobulins in humans (10). However, IgE can bind to the high affinity receptor for IgE (FcεRI) on mast cells and basophils and to the low affinity IgE receptor (CD23) on B cells and antigen presenting cells. Upon recognition of allergens by cell-bound IgE the effector cells become activated to release inflammatory mediators, proteases and cytokines. The activation of inflammatory cells by IgE-allergen immune complexes thus leads to allergic inflammation and a variety of allergic symptoms such as allergic rhinoconjunctivitis, asthma, skin inflammation, food allergy and life-threatening anaphylactic shock. The term allergy has been coined by the Viennese pediatrician Clemens von Pirquet in order to describe exaggerated immune responses against *per se* harmless antigens (11). IgE-associated allergy, also termed immediate type allergy, is the most prevalent and important immunologically mediated hypersensitivity disease affecting approximately 30% of the population (12).

The first step in the development of allergy is allergic sensitization which is characterized by the production of IgE antibodies against allergens shortly after birth (13). The development of IgE sensitization in early childhood has been studied recently in great detail in population-based birth cohorts using micro-arrayed allergen molecules (14, 15). These studies have analyzed in birth cohorts the development of IgE sensitization to a large number of respiratory and food allergen molecules by micro-array technology during the first two decades of life (16–23). According to these studies it seems that there is a time window early in life during which allergic sensitization can occur (24), whereas adult allergic patients do not change their IgE reactivity profiles any more (25). In several studies it was observed that the percentages of sensitized children increase during the first years of life, but it is not clear whether this is due to the development of “new sensitizations” during the first years or whether it is related to the ability to detect allergen-specific IgE antibodies in serum and plasma during this period.

**Figure 1:**
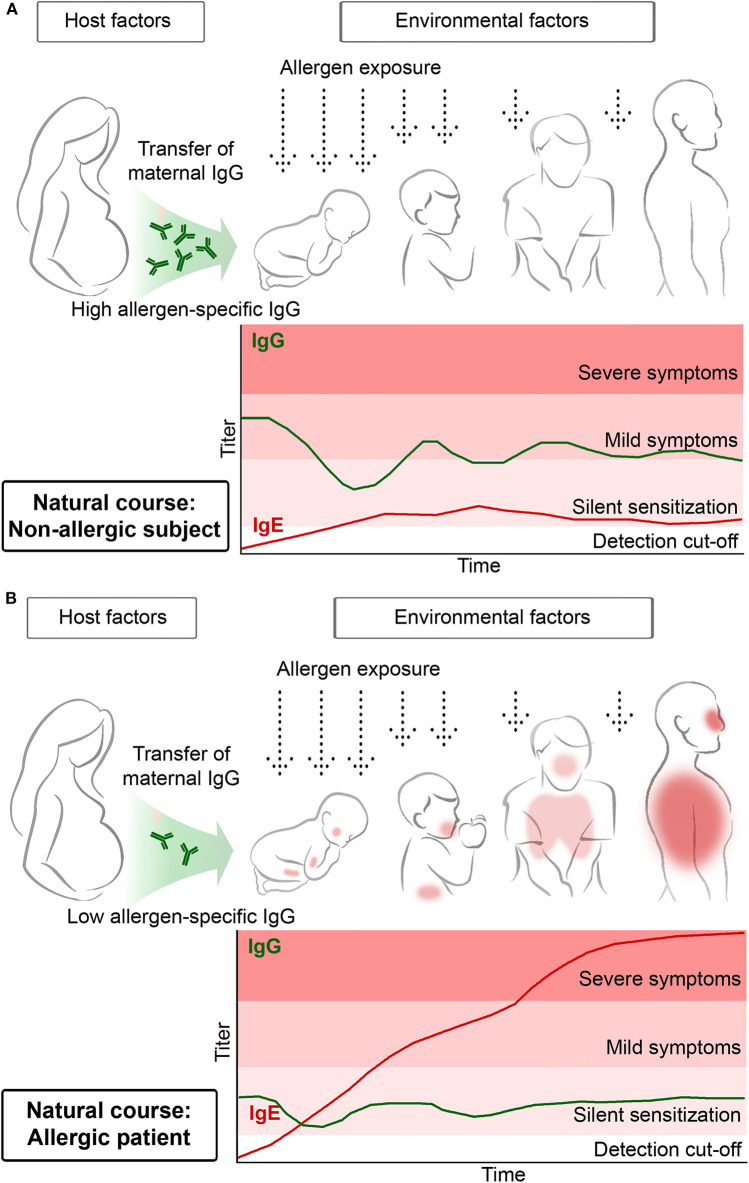
Transfer of high maternal allergen-specific IgG antibody levels may protect the off-spring from becoming sensitized and developing allergen-specific IgE antibodies **(A)** whereas low maternal allergen-specific IgG levels may predispose for allergic sensitization of the off-spring **(B)**.

A recent study observed that IgE sensitization rates were lower in children from mothers transferring higher levels of allergen-specific IgG antibodies by cord-blood to their children than in children whose mothers transmitted lower levels of specific IgG antibodies (Figure 1) (26). In the latter study, allergen-specific IgG antibodies of maternal origin could be traced in the children up to 6 months of life. Assuming that these IgG antibodies have a protective effect one could suggest that the first few months in life are the most critical period for allergic sensitization. This assumption is also supported by other studies reporting that children born shortly before pollen seasons became more frequently sensitized to pollen allergens than children born directly after cessation of the pollen season (27). In fact, the definition of the early time window during which allergic sensitization occurs is of great importance when considering preventive allergen-specific vaccination strategies.

Another important lesson learned from birth cohort studies is that repeated allergen contact may be needed to boost allergen-specific IgE production to certain levels so that clinically silent IgE sensitization can proceed toward allergic symptoms. This is indicated in Figure 1 by denoting that early in life IgE antibody production without symptoms (i.e., silent sensitization) may precede the development of allergic symptoms. In this context, two birth cohort studies should be mentioned. Westman et al. noted that at 4 years of life 12.5% of Swedish children had IgE antibodies against the major birch pollen allergen Bet v 1 but only 2.5% had allergic symptoms. This changed considerably when the children had become 16 years of age. Then, 25.4% had Bet v 1-specific IgE antibodies and the majority (i.e., 17.8%) had symptoms of birch pollen allergy (17). In a birth cohort study investigating the development of grass pollen allergy Westman et al. had similar results. At 4 years of age the vast majority of children with IgE antibodies against grass pollen allergens did not yet have symptoms whereas at the age of 16 almost 50% of IgE-positive children had developed symptoms (23). Furthermore, progression of mild symptoms toward severe symptoms later in childhood is common, for example progression of rhinitis toward asthma, and it was found that progression of clinically silent IgE sensitization to allergic symptoms can be predicted earlier in life based on IgE levels against allergen molecules (17, 21).

### Features of AIT

The first AIT study was performed by Leonard Noon in 1911 and demonstrated that immunization with grass pollen allergen extract improved symptoms of grass pollen allergy (28). In his classic paper, Noon entertains the idea of performing active vaccination for the treatment of allergy by referring to earlier work by Dunbar, who showed in 1903 that one can reduce symptoms of allergy with anti-sera raised against pollen allergens and thus showed that passive immunization is a possible treatment approach for allergy (29). We have summarized the development of AIT from past to presence in a recent review article which highlights important steps (30). They include the demonstration that the induction of allergen-specific IgG antibodies in the course of AIT blocks IgE binding to allergens and thus all the downstream effects of allergen-IgE immune complexes on activating inflammatory immune cells. The importance of the induction of allergen-specific IgG blocking antibodies has been demonstrated already very early by showing that passive transfer of IgG from AIT-treated patients can suppress allergic skin inflammation (31) up to a recent elegant study showing that passive administration of allergen-specific recombinant IgG_4_ antibodies is effective for treatment of cat allergy (32). Those forms of AIT, which robustly induce allergen-specific IgG, as demonstrated in clinical studies, are most commonly used and considered as effective treatment, in particular subcutaneous AIT based on adsorbed allergens or allergen derivatives. The induction of allergen-specific IgG is considered as a major mechanism of AIT (5, 33) and thus AIT can be considered as a form of therapeutic vaccination. The major reason why AIT is not fully developed as treatment for allergy is that current AIT is still based on natural allergen extracts which are often of poor quality (34). Accordingly, allergen extract-based AIT is often only partly effective (35, 36) and may induce side effects in allergic patients because allergen administration to an allergic subject can induce immediate and late phase allergic reactions (37, 38). Furthermore, cumbersome treatment schedules are needed to avoid side effects which strongly reduce patient's compliance (39). Therefore, molecular forms of AIT have been developed (40, 41). Molecular AIT forms are based on the precise knowledge of the disease-causing allergens of which many have been characterized in great detail by molecular cloning in the last 30 years (42).

### Molecular Forms of AIT

**Figure 2:**
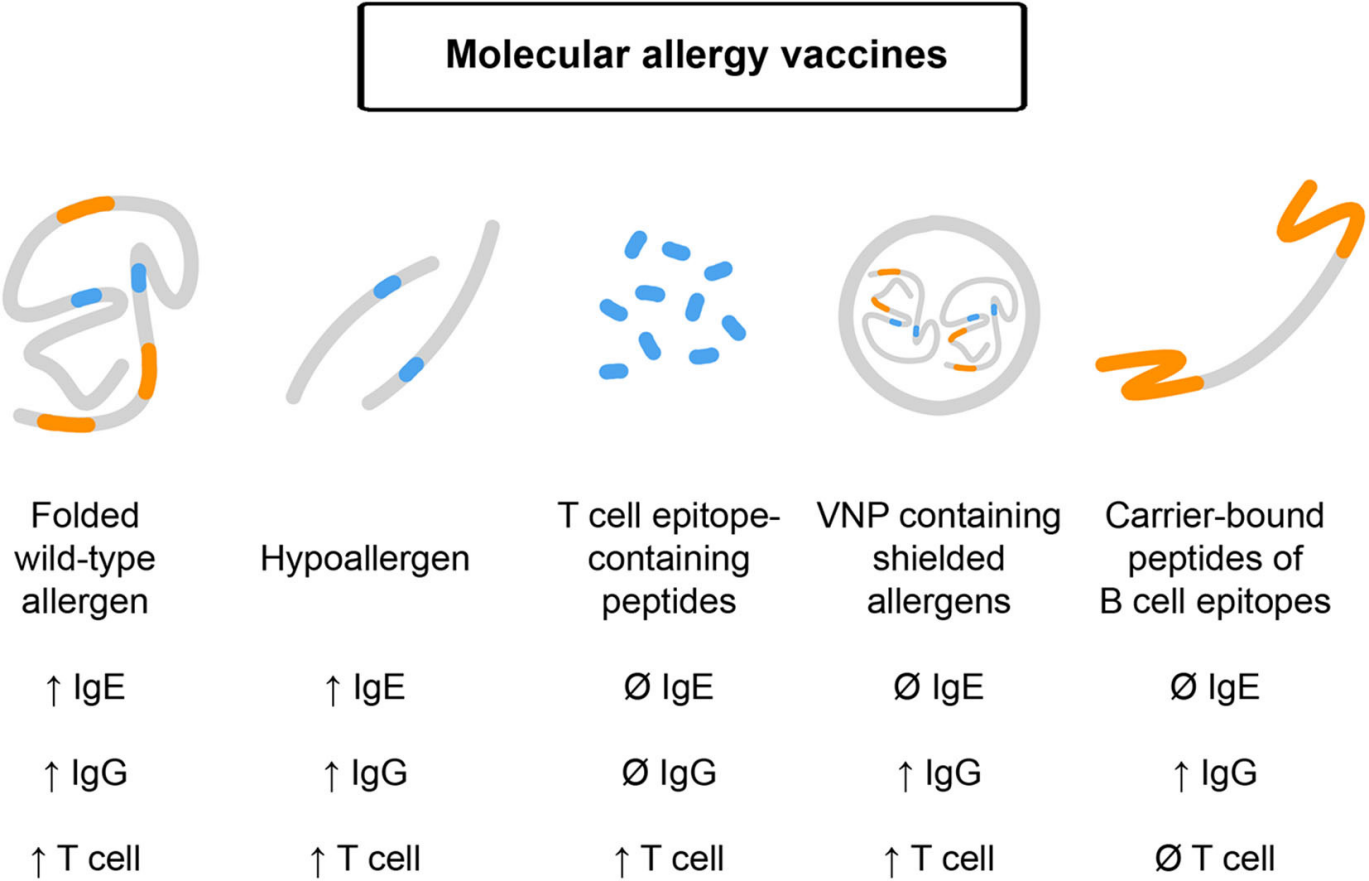
Molecular allergy vaccines which may be used for prophylactic vaccination and their features. Effects on IgE, IgG, and T cell responses upon administration are indicated. From left to right: Folded, wildtype-like recombinant allergens contain allergen-specific IgE, IgG, and T cell epitopes and may boost these responses upon immunization. Recombinant hypoallergens show reduced IgE reactivity but upon vaccination may induce allergen-specific IgE, IgG, and T cell responses. T cell epitope-containing peptides lack IgE and IgG reactivity and accordingly target T cells without inducing IgE or IgG responses. Virus-like nanoparticles can be produced to contain shielded allergens lacking IgE reactivity but may induce IgG and T cell responses. B cell epitope-based peptide carrier vaccines lack allergen-specific IgE and T cell reactivity and induce allergen-specific IgG responses without boosting allergen-specific IgE and T cell responses.

In this section, we will discuss different molecular forms of AIT, which could be pursued in clinical trials for preventive allergen-specific allergy vaccination (Figure 2). Please note that we have limited the molecular AIT forms to be considered for prophylactic vaccination to those, which have already been tested in clinical trials in allergic patients and for which immune responses obtained in humans upon vaccination have been studied (9, 41).

### Folded Wildtype-Like Recombinant Allergens

Folded wildtype-like recombinant allergens which mimic all properties of the naturally occurring allergens are produced for more than 30 years according to the DNA sequences of allergens. The wildtype-like recombinant allergens contain the IgE and T cell epitopes of the corresponding natural allergens. It is known from subcutaneous injection AIT (SCIT) trials performed with a mixture of recombinant grass pollen allergens and with recombinant major birch pollen allergen Bet v 1 that vaccination induces allergen-specific IgG antibodies which inhibit IgE binding to the allergen (43, 44). However, immunization with wildtype-like recombinant allergens also induces allergen-specific IgE antibody responses leaving the concern that they may boost allergen-specific IgE responses if they are used for secondary preventive vaccination or that they may even induce IgE sensitization if they are used for primary preventive vaccination in not yet sensitized subjects. This concern may be mitigated by the fact that studies showing that AIT can reduce the progression of allergic rhinitis to asthma in children were conducted by SCIT with natural allergens (45). Results from trials using sublingual immunotherapy (SLIT) with natural allergen extracts to prevent the progression of clinically silent IgE sensitization to allergic symptoms have been inconclusive (46, 47). The reason for this may have been that SLIT approaches are rather ineffective in inducing allergen-specific IgG responses as compared to SCIT (48). Accordingly, SCIT with folded wildtype-like recombinant allergens may, in principle, be considered for preventive vaccination but other molecular AIT forms with reduced risk for allergic sensitization may be preferred.

### Recombinant and Synthetic Hypoallergens

Recombinant and synthetic hypoallergens are hypoallergenic allergen derivatives with reduced IgE reactivity but preserved T cell epitopes (49). Hypoallergens differ from the corresponding wild-type allergens by modifications of their structure reducing their IgE reactivity as compared to the wildtype allergens. Thus, they have a reduced ability of inducing IgE-mediated allergic inflammation. However, the primary sequence of hypoallergens is almost unaltered in comparison to the wildtype allergens so that sequential T cell epitopes remain preserved and they can induce allergen-specific T cell activation. Already in the first clinical trial which has been performed with recombinant hypoallergenic allergen derivatives of the major birch pollen allergen Bet v 1, it has been shown that vaccination with such derivatives induces allergen-specific IgG blocking antibodies (50). However, immunization with hypoallergens can also boost allergen-specific IgE responses and due to the preservation of allergen-specific T cell epitopes may induce late-phase, T cell-mediated side effects in allergic patients (51).

Recombinant hypoallergenic fragments of Bet v 1 have been recently evaluated in a double-blind, placebo-controlled clinical study performed in non-allergic subjects for a period of 2 years (52). It was found that vaccination with recombinant Bet v 1 fragments induced continuously growing IgG responses which could prevent allergic patients' IgE binding to Bet v 1. Thus, this vaccine induced a protective IgG response. Although the induction of allergen-specific IgE responses was noted in subjects receiving recombinant Bet v 1 fragments, no allergic sensitization occurred because skin tests performed with Bet v 1 remained negative which may be attributed to the much higher induction of blocking IgG as compared to IgE. Accordingly, recombinant hypoallergenic allergen derivatives may be considered for prophylactic vaccination and there is evidence that vaccination of non-allergic subjects is safe. Yet the concern remains that also hypoallergens may induce allergen-specific IgE responses and thus allergic sensitization and that they may expand allergen-specific T cell responses due to the fact that allergen-specific T cell epitopes are preserved in these derivatives.

### T Cell Epitope-Containing Peptides

Allergen-derived T cell epitope-containing peptides are relatively short synthetic peptides of ~12–20 amino acids in length comprising T cell epitopes without any IgE reactivity (53). From AIT trials performed with such peptides it is known that depending on the applied dose and regimen they may induce T cell activation and eventually T cell tolerance (54, 55). Due to the fact that these peptides are very short they do not induce allergen-specific IgG blocking antibodies and hence will be not useful for prophylactic vaccination. However, they may be used for allergen-specific primary prevention by the induction of T cell tolerance (56). It may be envisaged that such peptides could be injected or even become applied by the oral route to induce profound tolerance of the adaptive immune system but this cannot be considered as prophylactic vaccination which should induce protective allergen-specific IgG antibodies. Challenges for approaches using T cell epitope-containing peptides for primary tolerance induction are to combine mixtures of peptides comprising all relevant allergens and MHC diversity of the subjects to be treated and the development of protocols for administration ensuring robust and sustained tolerance induction.

### Virus-Like Nanoparticles (VNP) Containing Shielded Allergens

In the first type of virus-like particle approaches allergen molecules were coupled chemically (57–59) and also by specific linker systems to virus-like particles produced by recombinant expression (60). This procedure yielded vaccines with reduced allergenic activity and ability to induce allergen-specific IgG responses. A vaccine consisting of a peptide derived from the major house dust mite allergen Der p 1 coupled to virus-like particles even has been subjected to clinical testing in non-allergic subjects and was found to induce allergen-specific IgG responses (61). However, the virus-like particle coupled allergens appeared as random conjugates and might even occur as large oligomers. Thus, they were difficult to produce under controlled conditions, which is required for clinical use and drug development. Recently, an alternative approach for engineering virus-like nanoparticles (VNP) was reported. In the VNPs, allergen-encoding cDNA is fused to virus-encoding DNA (Matrix protein, p15MA) (62) or to a glycosyl-phosphatidyl inositol anchor acceptor sequence (63) to be expressed either inside or outside of VNPs, respectively (64). In a mouse model of mugwort pollen allergy (65) such particles were successfully used for prophylactic vaccination (66) but so far there is no experience with VNPs in clinical AIT studies in patients and this approach therefore needs to be further developed.

### Carrier-Bound B Cell Epitope-Containing Peptide Vaccines

Carrier-bound B cell epitope-containing peptides are a further improvement of recombinant hypoallergens (38, 67, 68). In order to reduce side effects due to activation of allergen-specific T cells, allergen-specific T cell epitopes were reduced as much as possible and replaced by carrier proteins which are not derived from allergens but from viruses. Virus-derived proteins chosen were from rhinovirus and lastly from hepatitis B virus (HBV) with the intention of obtaining not only T cell help but also of eventually inducing a useful antiviral immunity (69, 70). The currently most advanced candidate vaccines use HBV-derived PreS protein as a carrier protein to which non-allergenic peptides derived from the IgE binding sites of allergens are fused (71–74). Thus, carrier-bound B cell epitope-containing vaccines can focus blocking IgG antibodies toward the IgE binding sites of allergens. The vaccine candidates can be produced in consistent quality satisfying GMP standards required for modern vaccine production because they can be expressed as recombinant fusion proteins in *Escherichia coli* in large quantities in a very cost-effective manner. Carrier-bound B cell epitope-containing peptide vaccines have been developed for several important allergen sources including pollen from trees, grasses, weeds, cats, house dust mites to name a few (68). These molecules have been characterized regarding their structural and immunological features *in vitro* and *in vivo* in animal models regarding their ability to induce IgG antibody responses which can block allergic patient's IgE binding to the natural allergens (75). Thus, the technology seems to be applicable to all known allergen sources. The carrier-bound B cell epitope-containing peptide vaccine made for grass pollen allergy, termed BM32 has been characterized best (73) and several clinical trials have been conducted or are ongoing with BM32 (76–78). Table 1 provides an overview of the clinical studies with BM32 or components thereof. In particular, the table displays the clinical trial numbers by referring to the official clinical trial database, the design of the studies and major findings made in these studies together with references to papers describing the clinical results.

**Table 1:** Clinical trials with recombinant B cell epitope-based peptide carrier vaccines.

**Name of the study**	**Designation in the ClinicalTrials.gov database of privately and publicly funded clinical studies conducted around the world**	**No of patients**	**Design**	**Major findings**	**References**
Skin test study of BM32	NCT01350635	60	Interventional, non-randomized, open-label	BM32 does not induce immediate or late-phase allergic skin inflammation and may be safe for vaccination	(76)
Phase II: Safety and dose finding trial of BM32 in subjects suffering from grass pollen allergy	NCT01445002	70	Prospective, randomized, double-blind, placebo-controlled, single center. Pollen exposure chamber	BM32 is well-tolerated; reduced allergic symptoms upon provocation with grass pollen by inducing allergen-specific IgG blocking antibodies. BM32 does not boost allergen-specific IgE production	(77)
Phase II field study of grass pollen allergy vaccine BM32	NCT01538979	181	Prospective, randomized, double-blind, placebo-controlled, parallel-group field study. One baseline year followed by 2 years of treatment	Injections of BM32 induced allergen-specific IgG, improved clinical symptoms of grass pollen allergy over two seasons and were well-tolerated. The optimal dose for BM32 was determined to be 20 μg per BM32 component/injection	(78)
Effect of different pre-seasonal BM32 dosings on the induction of a protective allergen-specific IgG response	NCT02643641	130	Prospective, randomized, double-blind, placebo-controlled, mono-centric, combination of pollen chamber and field study	Five injections of a mix of 20 μg of each BM32 component induced the best blocking IgG antibody response compared to three and four injections	
Study to evaluate induction of HBV virus neutralizing antibodies using VVX001 (i.e., BM325)	NCT03625934	84	Double-blind, randomized, placebo-controlled, multicenter study. Evaluation of the effects of VVX001 (i.e., BM325) to elicit a protective IgG immune response in vaccine naive subjects, in subjects who failed to demonstrate seroconversion after treatment with a licensed hepatitis B vaccine and in patients chronically infected with HBV	Ongoing Besides HBV-related endpoints, the study will provide information about safety in non-allergic subjects, induction of allergen-specific IgG responses and IgE sensitization capacity	

Starting with the safety evaluation of BM32 by skin testing (76) and in the subsequent AIT studies (77, 78), it was confirmed that BM32, due to abolished IgE reactivity, did not induce immediate type allergic reactions and there were only few and mild late-phase reactions due to the reduction of allergen-derived T cell epitopes. BM32 induced high levels of allergen-specific IgG antibodies which inhibited IgE binding to the grass pollen allergens and grass pollen allergen-induced basophil activation, immediate type allergic inflammation and allergen-induced T cell activation. Of note, much fewer injections of BM32 were equally effective compared to multiple injections with allergen extract-based vaccines (79). Patients tolerated high doses of BM32 so that few (i.e., 3–5) pre-seasonal injections in the first treatment year were sufficient to build up a strong blocking IgG antibody response and achieving a reduction of grass pollen allergen-induced symptoms of >25% over placebo (78). Importantly, it was noted that the allergen-specific blocking IgG response could be boosted to the original levels by only one booster injection (78). Allergen-specific IgG induced in the vaccinated patients not only reduced symptoms of grass pollen allergy but also prevented boosts of allergen-specific IgE production upon grass pollen allergen exposure during the pollen season, which led to a reduction of allergen-specific IgE levels in the treated patients (78).

Three more important observations were made in the clinical trials with BM32: First, BM32 induced allergen-specific IgG and in particular a continuously growing allergen-specific IgG_4_ response without boosting allergen-specific IgE as it is observed for all other AIT vaccines (80). Second, BM32 did not boost T cell and inflammatory cytokine responses specific for grass pollen allergens and thus seems to have no priming effect on allergen-specific T cell responses (80). Third, it was found that BM32 induced also IgG antibodies against HBV, which blocked the infection of *in vitro* cultured liver cells and thus it seems that BM32 also protects against HBV infection (81). For this reason there is currently another clinical study ongoing which investigates if BM325, a component of BM32, can induce protective HBV immune responses in subjects who have not been vaccinated against HBV and in subjects who belong to the 10–20% of non-responders to currently used S protein-based HBV vaccines (Table 1; ClinicalTrials.gov: NCT03625934). Furthermore, this trial also investigates if BM325 induces a therapeutic anti-HBV immune response in patients with chronic HBV infections. This study is interesting because it has enrolled subjects who are not allergic to grass pollen and it will thus be possible to study if BM325 is safe in non-allergic individuals and does not induce allergic sensitization. At present, BM32 has been evaluated in several phase II studies and an optimal dose and vaccination protocol has been established so that the vaccine is ready for a phase III trial which may provide the data required for the registration of the vaccine in Europe.

Due to the encouraging results obtained for BM32 in the clinical studies and some unique features which identify this vaccine as an excellent candidate for prophylactic vaccination we are considering BM32 and carrier-bound B cell epitope-containing peptide vaccines as suitable candidates for prophylactic vaccination against allergy. Next, we will briefly summarize and discuss these characteristics in light of potential use for preventive allergen-specific vaccination.

## Unique Features of Recombinant B Cell Epitope-Based Peptide Carrier Vaccines Predisposing Them for Preventive Allergen-Specific Allergy Vaccination

The recombinant B cell epitope-based peptide carrier vaccine for AIT of grass pollen allergy, BM32, has undergone extensive evaluation in clinical studies (Table 1). In the AIT studies, patients tolerated high doses of BM32 without experiencing severe immediate or late-phase allergic reactions. Few doses (i.e., 3–5 subcutaneous injections) induced allergen-specific IgG antibody responses directed toward IgE epitopes of the natural allergens. BM32 thus can focus blocking IgG responses against the epitopes on natural allergens involved in allergic sensitization. Due to replacement of allergen-specific T cell epitopes by carrier-specific T cell epitopes, BM32 showed a strongly reduced or no stimulation of allergen-specific T cell responses (80) and One may therefore expect that upon preventive vaccination early in life the vaccine will not prime T cells for allergic sensitization but induce allergen-specific IgG. Unlike all other allergen extract-based AIT vaccines, BM32 did not boost allergen-specific IgE responses and it might be assumed that the vaccine will not boost allergen-specific IgE responses when used for secondary preventive vaccination to prevent the transition of clinically silent sensitization toward allergic symptoms. Furthermore, it is likely that the vaccine will not induce allergic sensitization when used for primary preventive vaccination in non-allergic subjects. Only few vaccinations of BM32 were needed to induce strong allergen-specific blocking IgG response, which could be boosted by a single injection. Thus, it should be possible to induce in mothers or children early after birth a robust basic blocking IgG response, which can be boosted by single injections whenever needed. It has been shown that due to the use of the HBV-derived PreS protein as carrier protein in BM32, the latter induced also a protective immune response against HBV infections. Thus, the vaccine may not only be useful for prophylactic allergy vaccination but also for HBV vaccination.

### Recombinant B Cell Epitope-Based Peptide Carrier Vaccines for Secondary and Primary Preventive Allergy Vaccination

**Figure 3:**
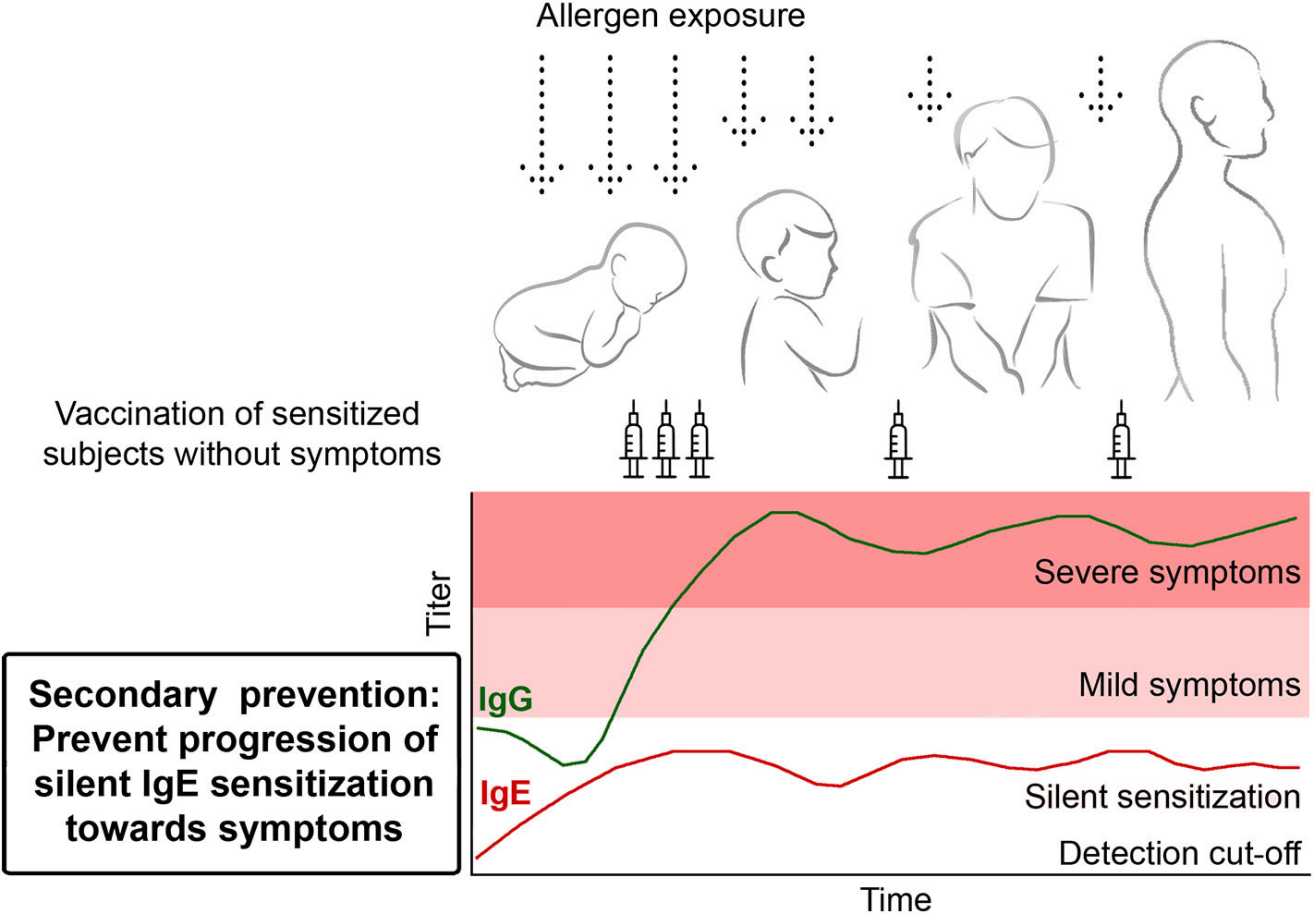
Secondary prevention. Prophylactic vaccination to prevent the transition from clinically silent IgE sensitization to the development of allergic symptoms. Children showing allergen-specific IgE reactivity are vaccinated in order to induce and maintain the production of blocking allergen-specific IgG antibodies to prevent the development of allergic symptoms.

**Figure 4:**
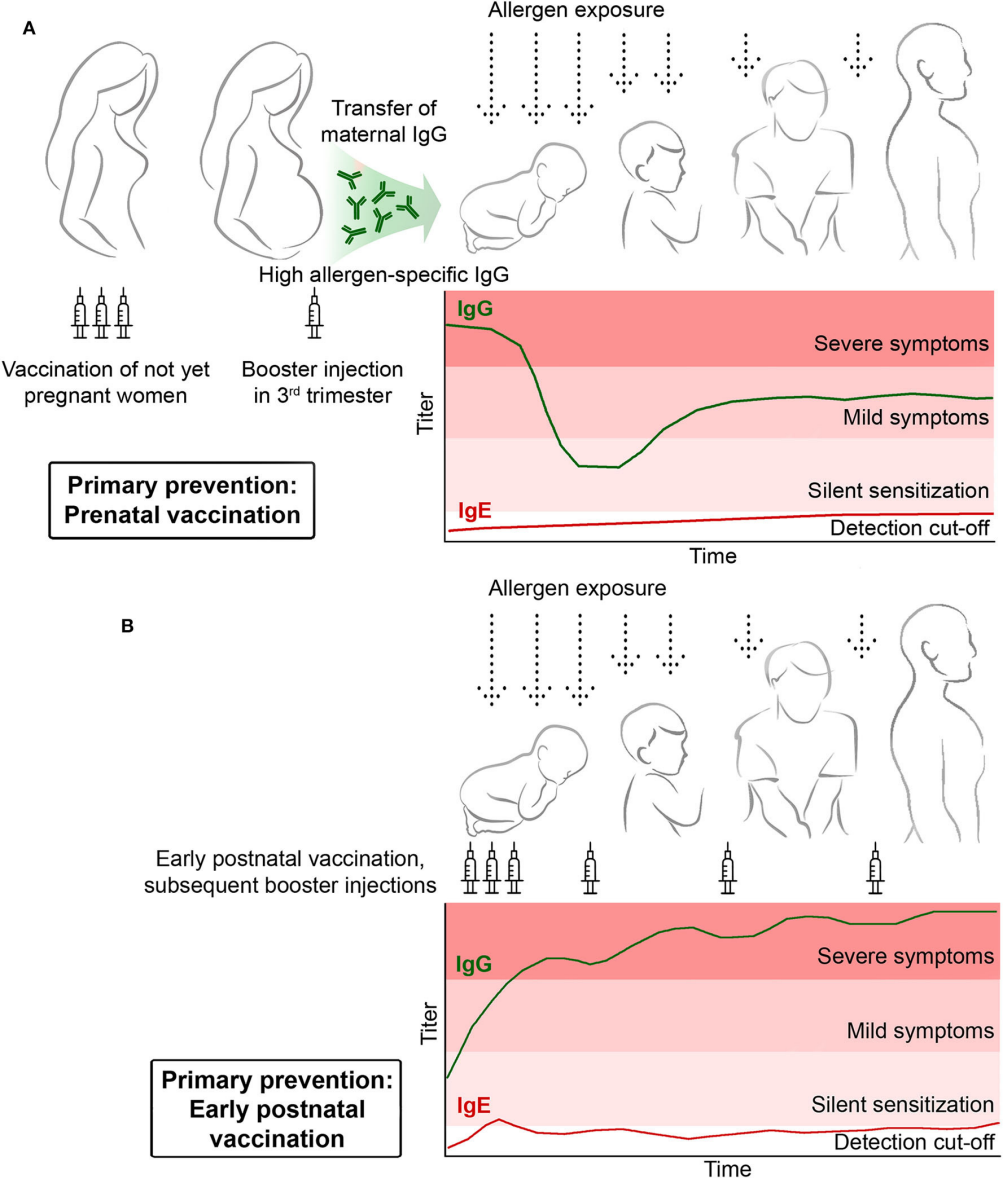
Primary prophylactic allergy vaccination. **(A)** Pre-natal vaccination of mothers should induce the basic production of allergen-specific blocking IgG antibodies which are then increased by a booster injection administered in the third trimester to transfer high levels of protective allergen-specific IgG to the child to prevent allergic sensitization postnatally. **(B)** Early post-natal prophylactic vaccination. Children receive early postnatal allergy vaccination to build up and maintain a protective allergen-specific IgG response to prevent allergic sensitization.

Here we consider three possible scenarios for prophylactic allergy vaccination (Figures 3, 4), which prevent either the progression of clinically silent IgE sensitization toward symptoms of allergy or even allergic sensitization. Together these strategies may allow eradicating the occurrence of severe forms of allergy on a population basis. Pre-requisites for these approaches are that the most important allergen molecules (i.e., most frequently recognized allergens with high allergenic activity) can be defined for a given population which is intended to be vaccinated and that recombinant B cell epitope-based peptide carrier vaccines can be formulated for these allergen molecules.

The analysis of IgE sensitization profiles toward comprehensive panels of micro-arrayed allergen molecules in population-based birth cohorts has shown that a handful of important allergen molecules can be defined for the formulation of such prophylactic vaccines (22). For this purpose it will only be necessary to define the molecular IgE sensitization profiles for different populations and countries in the world with an already existing technology of molecular diagnosis (14, 82). For many important respiratory allergen molecules, recombinant B cell epitope-based peptide carrier vaccines have already been pre-clinically characterized and it seems that the technology is broadly applicable to all types of allergens including also food and venom allergens (68). Nevertheless, we have focused in this article on inhalant allergies for several reasons: First, inhalant allergies are more than 10-fold more prevalent than food allergies and it will therefore be very difficult to conduct preventive AIT studies because very large numbers of study subjects will be needed to visualize if an allergen-specific preventive intervention is effective. Second, most of the progress regarding molecular immunotherapy strategies has been made in the field of inhalant allergies whereas relatively few candidate molecules are available for food allergy. Third, class 1 food allergens contain often sequential IgE epitopes and it may be technically more difficult to create non-sensitizing allergen derivatives for prevention. However, the strategy described in our perspective article will be fully applicable also for food allergy.

Since the underlying mechanisms of action of different recombinant B cell epitope-based peptide carrier vaccines have been shown to be identical (i.e., induction of allergen-specific blocking IgG responses), it will be easy to formulate the individual components separately and then mix them by taking into account the IgE recognition profiles of the different populations. The technology applied for BM32 currently is based on the adsorption of the four individual components onto aluminum hydroxide followed by mixing of the four adsorbates (73). Accordingly, such mixes can be formulated also for other components from unrelated allergen sources with this technology. Furthermore, it should be possible to combine peptides from different allergen molecules in single fusion proteins, which should allow reducing the number of components needed for allergy vaccines, which protect against several different allergen sources.

However, for many countries few important allergen sources can already be defined for prophylactic allergy vaccination. For example, birch pollen and cat allergies dominate in Scandinavia and Russia (17, 20, 83). A prophylactic birch pollen allergy vaccine would require only one allergen component, i.e., the major birch pollen allergen, Bet v 1 and a cat vaccine would be based primarily on the major cat allergen, Fel d 1. House dust mite allergy is by far the most important allergy affecting more than 20% of the population in Asia, Australia and many other parts of the world (84, 85). For house dust mites a cocktail of only 6 important allergen molecules has been defined, comprising Der p 1, Der p 2, Der p 5, Der p 7, Der p 21, and Der p 23 (86, 87). In Middle and Southern Europe and America grass pollen allergy dominates (88) and can be approached with BM32. In the Mediterranean area olive pollen and certain weeds such *Parietaria* are common requiring hyposensitization with Ole e 1 and Par j 1 as well as Par j 2 (89, 90), respectively. In Eastern Europe and parts of the USA ragweed dominates and mugwort pollen is important in Central Asia (91, 92).

**Figure 5:**
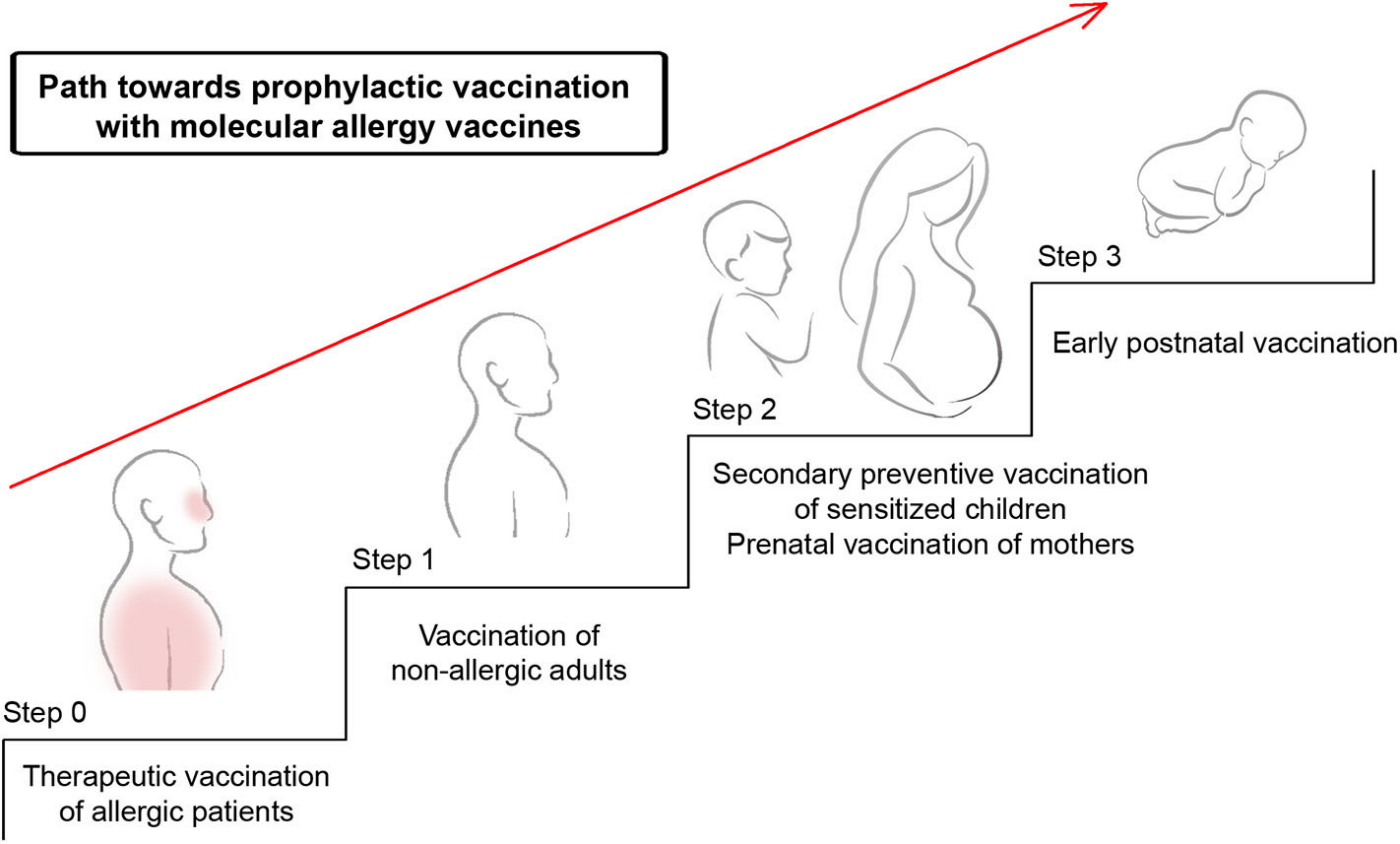
Possible path toward prophylactic vaccination with molecular allergy vaccines. Shown are systematic steps toward primary prophylactic allergy vaccination. Molecular allergy vaccines which have proven to be safe and to induce allergen-specific IgG responses without boosting IgE responses can be further evaluated in step 1 which comprises vaccination of non-allergic subjects to demonstrate that the vaccine is safe and does not induce allergic sensitization. In step 2, these vaccines will be evaluated for their ability to prevent the transition of clinically silent sensitization toward allergic symptoms by secondary prevention. Furthermore, it can be studied if prenatal vaccination of mothers can prevent the development of allergic sensitization in children by blocking IgG antibodies transferred from the mother to the child. As step 3, early postnatal vaccination may be considered to prevent the development of allergic sensitization in children.

With the available technology of recombinant B cell epitope-based peptide carrier vaccines it is thus immediately possible to explore concepts of prophylactic vaccination for major allergen source in certain of these countries/continents in proof of principle clinical trials. However, for each of the B cell epitope-based peptide carrier vaccines experience must be collected in therapeutic AIT trials as an initial step before prophylactic vaccination can be considered (Figure 5, Table 2). Below, we will discuss three different approaches for prophylactic allergy vaccination (Box 1: Secondary prevention, primary prevention).

**Table 2:** Possible path toward prophylactic vaccination with molecular allergy vaccines.

**Step**	**Type of study**	**Possible study endpoints**	**Possible favorable outcomes**
Step 0	Therapeutic vaccination of allergic patients	Safety. Induction of allergen-specific IgG blocking antibodies. Boosting of allergen-specific IgE responses. Induction of allergic sensitization Reduction of symptoms	Safe Induction of high levels of allergen-specific IgG without boosting allergen-specific IgE production. No induction of clinically relevant allergic sensitization. Reduction of symptoms
Step 1	Vaccination of non-allergic subjects	Safety. Induction of allergen-specific IgG blocking antibodies. Induction of allergic sensitization. Appearance of symptoms of allergy	Safe Induction of high levels of allergen-specific IgG inhibiting allergic patients IgE binding to allergens. No induction of clinically relevant allergic sensitization
Step 2	Secondary preventive vaccination of sensitized children	Safety. I Prevention of transition of mild allergic symptoms toward severe symptoms. Effects on symptoms II. Prevention of transition of clinically silent IgE sensitization to symptoms. Development of symptoms	Safe. I. Prevention of progression of mild toward severe symptoms II. Prevention of progression of clinically silent IgE sensitization to symptoms of allergy
Step 2	Prenatal vaccination of mothers	Safety. Induction of allergen-specific IgG in mothers. Prevention of allergen-specific IgE sensitization in off-springs	Safe Induction of allergen-specific IgG in mothers. Prevention of allergen-specific IgE sensitization in off-springs
Step 3	Early postnatal primary preventive vaccination of not yet sensitized children	Safety. Prevention of allergen-specific IgE sensitization. Induction of allergen-specific IgG	Safe Induction of allergen-specific IgG capable of inhibiting IgE binding to allergen. No induction of clinically relevant allergic sensitization. Prevention of allergen-specific IgE sensitization

### Secondary Prevention: Prevention of the Progression of Silent IgE Sensitization Toward Symptoms

Data obtained by longitudinal testing of children in population-based birth cohorts with micro-arrayed allergen molecules have shown that at the age of 4–6 years the majority of children have only clinically silent IgE sensitizations to respiratory allergens that later in life progress to symptoms of allergy (17, 23). IgE levels associated with clinically silent IgE sensitization are usually low and it seems that threshold IgE levels for silent sensitization and symptomatic allergy can be defined (83). Furthermore, these studies have demonstrated that allergen-specific IgE reactivity patterns and levels measured early in life (i.e., at ages of 4–6 years) are useful to predict the progression toward symptoms of allergy (17, 22). Accordingly, these birth cohort studies have indicated that one can identify children with clinically silent sensitization at early age, who have an increased risk for becoming allergic later in life. It is thus possible to identify children to prevent the progression of silent IgE sensitization toward allergic symptoms by secondary preventive allergen-specific vaccination (Figure 3). Practically, it can be imagined that such children are identified with serological tests detecting IgE responses to a comprehensive panel of micro-arrayed allergen molecules and to vaccinate these children with B cell epitope-based peptide carrier vaccines against the relevant allergens. Proof of principle clinical studies could be double-blind, placebo-controlled studies, in which one group of children receives active vaccination whereas a control group is treated with placebo. Taken into consideration the knowledge that >3 years treatment is required to achieve long-term clinical benefits by AIT after discontinuation of treatment (93) one could envisage that the prototype studies would comprise 3 years of treatment and a follow-up period of another 2 years. Already during treatment and in the follow up-period one would record the appearance of allergic symptoms in the two groups as clinical endpoint, safety and measure the induction of allergen-specific IgG blocking antibodies and IgE sensitization as immunological surrogate markers (Table 2). The advantage of the B cell epitope-based peptide carrier vaccines is that only few vaccinations are necessary to build up and maintain the blocking IgG antibody response. Furthermore, it has been shown for the grass pollen allergy vaccine BM32 that vaccination-induced IgG reduces the boost of allergen-specific IgE antibodies during pollen seasons (78). One may therefore hope that this will also occur in a secondary prevention study and that allergen-specific IgE levels will be kept below the threshold level for symptoms whereas allergen-specific IgG is increased in vaccinated children (Figure 3).

However, one needs to bear in mind that the approach of secondary prevention will require continuous treatment during life because IgE sensitization has already occurred in the target population and upon decline of protective IgG antibody levels allergen-specific IgE and associated symptoms may appear.

### Primary Prevention by Prenatal Maternal Allergen-Specific Vaccination

Several lines of evidence suggest that primary preventive allergen-specific vaccination against allergy can be achieved by maternal vaccination. First of all, it has been demonstrated in several experimental animal studies that maternal immunization can suppress allergic sensitization of the off-spring (94–97). Second, it was shown that that the protective effect is mainly mediated by the transfer of allergen-specific IgG antibodies in models of passive immunization (98–101). Third, there is evidence from clinical experience that AIT of pregnant women can protect the children from allergen-specific IgE sensitization (102) and IgG_1_ and IgG_4_ antibodies induced in pregnant women by AIT were found to be transmitted to the child and could be detected in cord blood (103). Finally, a recent study has indicated that high levels of natural maternal allergen-specific IgG antibodies may protect against allergic sensitization in the children when they were followed up until the age of 5 years in a birth-cohort study (26).

The concept of maternal vaccination is in fact quite well-accepted in the field of infectious diseases. Maternal vaccination is considered particularly in the field of influenza (104, 105), group B streptococcus diseases (106), pertussis (107), and several other infectious diseases as safe and effective (108–110).

Practically one could consider maternal vaccination with recombinant B cell epitope-based peptide carrier vaccines once it has been demonstrated that the vaccine does not induce allergic sensitization and is safe in non-allergic subjects but induces high levels of allergen-specific IgG blocking antibodies (Figures 4A, 5, Table 2). There is currently no reliable marker which would safely predict if children from certain parents will develop allergic sensitization to a particular allergen molecule. Therefore, proof of principle studies investigating if primary allergy prevention by maternal vaccination will not require pre-selection of the participating mothers. Such studies should be performed in populations in which sensitizations to certain allergens are very common and suitable vaccines for these molecules should be available. For example, preventive studies for grass pollen allergy could be performed with BM32 in Middle Europe, vaccination against birch pollen allergy could be done with a hypoallergenic Bet v 1 variant in Russia and Scandinavia and preventive vaccination against house dust mite allergy in large parts of Asia. Such proof of principle studies could enroll women with a wish of having children by administering to them a course of basic vaccinations to build up allergen-specific IgG responses and once they become pregnant, allergen-specific IgG could be boosted up with a single booster injection given in the third trimester. Such a proof of principle study could be conducted as double-blind, placebo-controlled study by having a group of mothers who receives active vaccination and one which receives placebo. The occurrence of IgE sensitization and of allergic symptoms in children followed up during the first few years (e.g., 3–5 years) would be important endpoints of the study (Table 2). The transmission of allergen-specific IgG from the mother to the child and the development of the child's own allergen-specific antibody responses could be measured by analyzing capillary blood samples using allergen micro-arrays as reported recently (26).

The major hope and expectation with the approach of maternal vaccination would be that it can prevent allergic sensitization of the child without requiring further vaccination of the children. However, this has to be demonstrated in proof of principle clinical studies.

### Primary Prevention by Early Post-natal Allergen-Specific Vaccination

In order to achieve primary allergy prevention by early postnatal vaccination, immunization needs to be done before allergic sensitization has taken place. Otherwise the approach would qualify only as a secondary preventive approach (Box 1). Thus, children would need to be vaccinated shortly after birth to build up a blocking allergen-specific IgG response to prevent allergic sensitization. In the case of prophylactic vaccines for infectious diseases the goal is to establish a specific B cell memory which is activated upon infection and will protect ideally life-long. Regarding allergy, the goal is to prevent allergic sensitization which according to birth cohort studies occurs only early in childhood whereas allergen contact later in life does not induce allergic sensitization. It therefore does not seem to be important to generate B cell memory which can be activated by allergen contact later in life but to keep protective allergen-specific antibodies high during early life when allergic sensitization occurs.

Box 1 **Definitions relevant to approaches for allergy vaccination.**

Therapeutic vaccination: Allergen-specific immunotherapy (AIT)

SCIT: Subcutaneous allergen-specific immunotherapy

SLIT: Sublingual allergen-specific immunotherapy

Secondary prevention: Prevention of progression of mild symptoms to severe disease

Secondary prevention: Prevention of progression of clinically silent sensitization to symptoms

Primary prevention: Prevention of IgE sensitization

Recombinant peptide-carrier allergy vaccine: Recombinant allergy vaccine consisting of a non-allergen-derived carrier molecule and non-allergenic peptides derived from the IgE binding sites of the allergen.

While there is evidence for a genetic predisposition for becoming sensitized toward particular allergen molecules depending on the HLA background (9, 65), there are currently no definitive markers which would allow to safely define children who are at risk of becoming sensitized against a particular allergen. Accordingly, there will be no possibilities to select children for a proof-of-principle study for primary preventive vaccination. However, the study would need to be performed in populations in which certain allergies are highly prevalent as has been described for maternal vaccination. In such a proof-of-principle study children would receive shortly after birth a course of vaccinations with B cell epitope-based peptide carrier vaccines made for allergens which are highly prevalent in the population to build up an allergen-specific IgG blocking antibody response (Figure 4B). This IgG response should be maintained by booster injections for a period of ~3 years which is the preferred time for AIT studies to build up sustained IgG responses. The study should include a placebo group receiving only adjuvant and thus would be designed as double-blind, placebo-controlled study (Table 2). During the 3 years of vaccination and during a follow up period of ~2 to 3 years thereafter children would be monitored to study the development of IgE sensitizations and allergic symptoms and to monitor allergen-specific IgG antibody responses in addition to a careful safety assessment (Table 2). Ideally, the study would show that primary preventive vaccination of the children is safe, induces an allergen-specific IgG blocking antibody response and specifically prevents allergic sensitization.

## A Possible Path Toward Preventive Allergen-Specific Allergy Vaccination

In Figure 5, we suggest a possible path toward prophylactic vaccination with molecular allergy vaccines which involves different steps to ensure safety and demonstration of feasibility. We argue that molecular allergy vaccines which are considered for prophylactic vaccination should be evaluated initially in therapeutic AIT studies in allergic patients (Figure 5, step 0). Such studies will provide information regarding the safety of these vaccines in allergic patients and it can be assessed if the vaccines induce desired allergen-specific blocking IgG responses with low or no boosting of allergen-specific IgE responses. Furthermore, it can be determined if the allergen-specific IgG antibodies indeed block allergic patients' IgE binding to the allergens and provide a beneficial clinical effect.

In a next or parallel step to therapeutic AIT, the vaccines should be evaluated in a clinical study in non-allergic adult subjects to investigate if the vaccine is also safe in non-allergic patients. In particular it has to be studied if the vaccine induces any relevant allergic sensitization as has been done in a recently published study (52) (Figure 5, step 1, Table 2). Allergen-specific IgG antibodies will be tested for their ability to block allergic patients' IgE binding to the allergen and the kinetics of their induction, their levels and how long they persist will be analyzed to facilitate the planning of preventive studies.

After step 0 and 1, data should be available to plan secondary preventive studies which are thought to investigate if vaccination can prevent the progression of clinically silent IgE sensitization toward allergic symptoms and/or the progression of mild symptoms such as rhinitis toward severe ones, e.g., asthma. These studies will benefit much from the experience collected in the therapeutic AIT studies because they represent a logic continuation of the therapeutic studies. In fact, it is quite likely that secondary prevention will be much more safe and effective than therapeutic AIT because these approaches will prevent the occurrence of severe allergic symptoms. Thus, one has to consider that it will be much more difficult to treat allergic subjects with already existing severe allergic symptoms than subjects with no or mild symptoms because side effects may be much less frequent, if any, in the latter group of subjects. Data from studies analyzing vaccination of non-allergic subjects will inform about the IgE sensitization capacity of the vaccines and hence will provide important information for secondary preventive approaches because any boosting of IgE responses in IgE sensitized subjects without clinical symptoms or in subjects with mild allergy would be unintended.

Furthermore, steps 0 and 1 will be important prerequisites for primary maternal vaccination because maternal vaccination will be performed ultimately in mothers who are allergic or not sensitized. Regarding vaccination of allergic mothers it is important that the safety of the vaccine in allergic patients has been demonstrated in AIT studies whereas for vaccination of non-allergic mothers it will be important to know that the vaccine does not induce relevant allergic sensitization.

Since early postnatal allergen-specific vaccination of not sensitized children involves very young children and requires experience with the intended vaccine regarding safety and lack of sensitization capacity we suggest to consider it as the last step (i.e., step 3) after therapeutic AIT studies (step 0), vaccination of non-allergic adults (step 1), secondary preventive vaccination of children (step 2) or maternal vaccination (step 2) (Figure 5, Table 2). Ideally, primary preventive allergy vaccination may already be achieved by maternal vaccination which would reduce the need for early post-natal preventive vaccination of children.

The indicated path toward prophylactic vaccination (Figure 5, Table 2) could be accelerated through the use of modern molecular allergy vaccines if tested for several important allergens in parallel. Furthermore, it will be helpful if vaccines are used which resemble the advantageous features of the B cell epitope-based peptide carrier vaccines and build up protective allergen-specific IgG antibody responses. A similar mode of action of vaccines may in fact reduce the number of clinical trials needed.

Important next steps will be to follow the path described in Table 2 with available B cell epitope-based peptide carrier vaccines, such as BM32, and to investigate in proof-of-principle studies if the proposed concept for prophylactic vaccination is indeed feasible.

## Conclusion

Prophylactic vaccines are available for infectious diseases but not yet for IgE-associated allergy, the most common immune mediated hypersensitivity disease. Allergen-specific immunotherapy (AIT) is an effective and disease-modifying treatment for allergy. It represents a therapeutic vaccination, which induces allergen-specific IgG antibodies blocking IgE recognition of allergens and subsequent allergic inflammation induced by IgE-allergen immune complexes. AIT is an economic and the only disease-modifying treatment with long-lasting effects even after discontinuation but currently only 10% of allergic patients receive AIT treatment. The broad application and further development of AIT is limited by the poor quality of natural allergens extracts. With the molecular characterization of the disease-causing allergens, new forms of molecular AIT have been developed of which B cell epitope-based peptide carrier vaccines have been evaluated in several clinical trials and were found to exhibit several characteristics which make them possible candidates for prophylactic allergy vaccination. We suggest a stepwise evaluation of these new molecular allergy vaccines in clinical trials to develop vaccines for secondary and primary vaccination against allergy. Secondary preventive allergy vaccination may prevent the development of severe allergic symptoms in allergic patients and thus may be considered more safe and effective than therapeutic vaccination of patients suffering already from severe allergic symptoms. Primary prevention of allergic sensitization may be achieved by maternal vaccination inducing blocking allergen-specific IgG antibodies that are transmitted by the placenta and may prevent allergic sensitization in the off-spring. Alternatively, primary preventive vaccination of not yet sensitized children early after birth may be considered. Preventive allergy vaccination with modern molecular allergy vaccines may be a possible mission that holds the promise for eradicating allergic diseases similar as has been achieved for infectious diseases.

## Author Contributions

RV and IT wrote the manuscript and designed the figures and tables. BK, RC, MC, MH, AKars, KR, AKara, MK, and WP critically read and revised the manuscript. All authors contributed to the article and approved the submitted version.

## Conflict of Interest

RV has received research grants from HVD Life Science, Vienna, Austria and Viravaxx, Vienna, Austria and serves as a consultant for these companies. MH serves as consultant for Biomay AG, Vienna, Austria. WP holds stocks of Biomay AG and receives honoraria from Novartis, Pfizer, and Roche. The remaining authors declare that the research was conducted in the absence of any commercial or financial relationships that could be construed as a potential conflict of interest. The reviewer AL declared a past co-authorship with one of the authors MH to the handling editor.

## Abbreviations

Ab: antibody
AIT: allergen-specific immunotherapy
APC: antigen presenting cell
GMP: Good Manufacturing Practice
HBV: hepatitis B virus
Ig: immunoglobulin
IL: interleukin
SCIT: subcutaneous immunotherapy
SLIT: sublingual immunotherapy
VNP: virus-like nanoparticles.

## References

[1] Piot P Larson HJ O'Brien KL N'kengasong J Ng E Sow S. Immunization: vital progress, unfinished agenda. Nature. (2019) 575:119–29. 10.1038/s41586-019-1656-7 31695203

[2] Rappuoli R Mandl CW Black S De Gregorio E. Vaccines for the twenty-first century society. Nat Rev Immunol. (2011) 11:865–72. 10.1038/nri3085 22051890 PMC7098427

[3] Andreano E D'Oro U Rappuoli R Finco O. Vaccine evolution and its application to fight modern threats. Front Immunol. (2019) 10:1722. 10.3389/fimmu.2019.01722 31404139 PMC6669413

[4] Ada G. Vaccines and vaccination. N Engl J Med. (2001) 345:1042–53. 10.1056/NEJMra011223 11586958

[5] Larché M Akdis CA Valenta R. Immunological mechanisms of allergen-specific immunotherapy. Nat Rev Immunol. (2006) 6:761–71. 10.1038/nri1934 16998509

[6] Eckl-Dorna J Villazala-Merino S Linhart B Karaulov AV Zhernov Y Khaitov M. Allergen-specific antibodies regulate secondary allergen-specific immune responses. Front Immunol. (2019) 9:3131. 10.3389/fimmu.2018.03131 30705676 PMC6344431

[7] Hofmaier S Hatzler L Rohrbach A Panetta V Hakimeh D Bauer CP. “Default” versus “pre-atopic” IgG responses to foodborne and airborne pathogenesis-related group 10 protein molecules in birch-sensitized and nonatopic children. J Allergy Clin Immunol. (2015) 135:1367–74.e8. 10.1016/j.jaci.2014.09.048 25458000

[8] Siroux V Lupinek C Resch Y Curin M Just J Keil T. Specific IgE and IgG measured by the MeDALL allergen-chip depend on allergen and route of exposure: The EGEA study. J Allergy Clin Immunol. (2017) 139:643–54.e6. 10.1016/j.jaci.2016.05.023 27464960

[9] Valenta R Karaulov A Niederberger V Gattinger P van Hage M Flicker S. Molecular aspects of allergens and allergy. Adv. Immunol. 138:195–256. 10.1016/bs.ai.2018.03.002 29731005

[10] Eckl-Dorna J Villazala-Merino S Campion NJ Byazrova M Filatov A Kudlay D. Tracing IgE-producing cells in allergic patients. Cells. (2019) 8:994. 10.3390/cells8090994 31466324 PMC6769703

[11] Münchener medizinische wochenschrift/24 July 1906: allergy by Clemens v. Pirquet, Vienna. MMW Munch Med Wochenschr. (1978) 120:474. 347265

[12] Anto JM Bousquet J Akdis M Auffray C Keil T Momas I. Mechanisms of the Development of Allergy (MeDALL): introducing novel concepts in allergy phenotypes. J Allergy Clin Immunol. (2017) 139:388–99. 10.1016/j.jaci.2016.12.940 28183433

[13] Kulig M Bergmann R Klettke U Wahn V Tacke U Wahn U. Natural course of sensitization to food and inhalant allergens during the first 6 years of life. J Allergy Clin Immunol. (1999) 103:1173–9. 10.1016/S0091-6749(99)70195-8 10359902

[14] Lupinek C Wollmann E Baar A Banerjee S Breiteneder H Broecker BM. Advances in allergen-microarray technology for diagnosis and monitoring of allergy: the MeDALL allergen-chip. Methods. (2014) 66:106–19. 10.1016/j.ymeth.2013.10.008 24161540 PMC4687054

[15] Bousquet J Gern JE Martinez FD Anto JM Johnson CC Holt PG. Birth cohorts in asthma and allergic diseases: report of a NIAID/NHLBI/MeDALL joint workshop. J Allergy Clin Immunol. (2014) 133:1535–46. 10.1016/j.jaci.2014.01.018 24636091 PMC4088262

[16] Hatzler L Panetta V Lau S Wagner P Bergmann RL Illi S. Molecular spreading and predictive value of preclinical IgE response to phleum pratense in children with hay fever. J Allergy Clin Immunol. (2012) 130:894–901.e5. 10.1016/j.jaci.2012.05.053 22841010

[17] Westman M Lupinek C Bousquet J Andersson N Pahr S Baar A. Early childhood IgE reactivity to pathogenesis-related class 10 proteins predicts allergic rhinitis in adolescence. J Allergy Clin Immunol. (2015) 135:1199–206.e11. 10.1016/j.jaci.2014.10.042 25528361 PMC6597345

[18] Custovic A Sonntag H-J Buchan IE Belgrave D Simpson A Prosperi MCF. Evolution pathways of IgE responses to grass and mite allergens throughout childhood. J Allergy Clin Immunol. (2015) 136:1645–52.e8. 10.1016/j.jaci.2015.03.041 25962900

[19] Posa D Perna S Resch Y Lupinek C Panetta V Hofmaier S. Evolution and predictive value of IgE responses toward a comprehensive panel of house dust mite allergens during the first 2 decades of life. J Allergy Clin Immunol. (2017) 139:541–9.e8. 10.1016/j.jaci.2016.08.014 27793411

[20] Asarnoj A Hamsten C Wadén K Lupinek C Andersson N Kull I. Sensitization to cat and dog allergen molecules in childhood and prediction of symptoms of cat and dog allergy in adolescence: A BAMSE/MeDALL study. J Allergy Clin Immunol. (2016) 137:813–21.e7. 10.1016/j.jaci.2015.09.052 26686472 PMC6597346

[21] Asarnoj A Hamsten C Lupinek C Melén E Andersson N Anto JM. Prediction of peanut allergy in adolescence by early childhood storage protein-specific IgE signatures: the BAMSE population-based birth cohort. J Allergy Clin Immunol. (2017) 140:587–90.e7. 10.1016/j.jaci.2016.12.973 28192142

[22] Wickman M Lupinek C Andersson N Belgrave D Asarnoj A Benet M. Detection of IgE reactivity to a handful of allergen molecules in early childhood predicts respiratory allergy in adolescence. EBioMed. (2017) 26:91–9. 10.1016/j.ebiom.2017.11.009 29221963 PMC5832567

[23] Westman M Åberg K Apostolovic D Lupinek C Gattinger P Mittermann I Andersson N. Sensitization to grass pollen allergen molecules in a birth cohort – Phl p 4 as early indicator of grass pollen allergy. J Allergy Clin Immunol. (2020) 145:1174–81.e6. 10.1016/j.jaci.2020.01.006 31954777

[24] Westman M Asarnoj A Hamsten C Wickman M van Hage M. Windows of opportunity for tolerance induction for allergy by studying the evolution of allergic sensitization in birth cohorts. Semin Immunol. (2017) 30:61–6. 10.1016/j.smim.2017.07.005 28789818

[25] Lupinek C Marth K Niederberger V Valenta R. Analysis of serum IgE reactivity profiles with microarrayed allergens indicates absence of *de novo* IgE sensitizations in adults. J Allergy Clin Immunol. (2012) 130:1418–20.e4. 10.1016/j.jaci.2012.06.028 22867692 PMC4578245

[26] Lupinek C Hochwallner H Johansson C Mie A Rigler E Scheynius A. Maternal allergen-specific IgG might protect the child against allergic sensitization. J Allergy Clin Immunol. (2019) 144:536–48. 10.1016/j.jaci.2018.11.051 30685457 PMC6689269

[27] Pearson DJ Freed DL Taylor G. Respiratory allergy and month of birth. Clin Allergy. (1977) 7:29–33. 10.1111/j.1365-2222.1977.tb01421.x 872354

[28] Noon L Prophylactic inoculation against hay fever. Lancet. (1911) 177:1572–3. 10.1016/S0140-6736(00)78276-6 13096152

[29] Dunbar WP Weiterer beitrag zur ursache und spezifischen heilung des heufiebers. DMW Dtsch Med Wochenschr. (1903) 29:149–52. 10.1055/s-0028-1138323

[30] Dorofeeva Y Shilovsky I Tulaeva I Focke-Tejkl M Flicker S Kudlay D. Past, presence and future of allergen immunotherapy vaccines. Allergy. (2020) 1–19. 10.1111/all.14300 32249442 PMC7818275

[31] Cooke RA Barnard JH Hebald S Stull A. Serological evidence of immunity with coexisting sensitization in a type of human allergy (hay fever). J Exp Med. (1935) 62:733–50. 10.1084/jem.62.6.733 19870445 PMC2133309

[32] Orengo JM Radin AR Kamat V Badithe A Ben LH Bennett BL. Treating cat allergy with monoclonal IgG antibodies that bind allergen and prevent IgE engagement. Nat Commun. (2018) 9:1421. 10.1038/s41467-018-03636-8 29650949 PMC5897525

[33] Shamji MH Durham SR. Mechanisms of allergen immunotherapy for inhaled allergens and predictive biomarkers. J Allergy Clin Immunol. (2017) 140:1485–98. 10.1016/j.jaci.2017.10.010 29221580

[34] Valenta R Karaulov A Niederberger V Zhernov Y Elisyutina O Campana R. Allergen extracts for *in vivo* diagnosis and treatment of allergy: is there a future? J Allergy Clin Immunol Pract. (2018) 6:1845–55.e2. 10.1016/j.jaip.2018.08.032 30297269 PMC6390933

[35] Mothes N Heinzkill M Drachenberg KJ Sperr WR Krauth MT Majlesi Y. Allergen-specific immunotherapy with a monophosphoryl lipid a-adjuvanted vaccine: reduced seasonally boosted immunoglobulin E production and inhibition of basophil histamine release by therapy-induced blocking antibodies. Clin Exp Allergy. (2003) 33:1198–208. 10.1046/j.1365-2222.2003.01699.x 12956739

[36] Chen K-W Zieglmayer P Zieglmayer R Lemell P Horak F Bunu CP. Selection of house dust mite–allergic patients by molecular diagnosis may enhance success of specific immunotherapy. J Allergy Clin Immunol. (2019) 143:1248–52.e12. 10.1016/j.jaci.2018.10.048 30445063

[37] Winther L Arnved J Malling H-J Nolte H Mosbech H. Side-effects of allergen-specific immunotherapy: a prospective multi-centre study. Clin Exp Allergy. (2006) 36:254–60. 10.1111/j.1365-2222.2006.02340.x 16499635

[38] Focke M Swoboda I Marth K Valenta R Developments in allergen-specific immunotherapy: from allergen extracts to allergy vaccines bypassing allergen-specific immunoglobulin E and T cell reactivity. Clin Exp Allergy. (2010) 40:385–97. 10.1111/j.1365-2222.2009.03443.x 20210812

[39] Kiel MA Röder E Gerth van Wijk R Al MJ Hop WCJ Rutten-van Mölken MPMH. Real-life compliance and persistence among users of subcutaneous and sublingual allergen immunotherapy. J Allergy Clin Immunol. (2013) 132:353–60.e2. 10.1016/j.jaci.2013.03.013 23651609

[40] Curin M Khaitov M Karaulov A Namazova-Baranova L Campana R Garib V. Next-generation of allergen-specific immunotherapies: molecular approaches. Curr Allergy Asthma Rep. (2018) 18:39. 10.1007/s11882-018-0790-x 29886521 PMC5994214

[41] Zhernov Y Curin M Khaitov M Karaulov A Valenta R. Recombinant allergens for immunotherapy: state of the art. Curr Opin Allergy Clin Immunol. (2019) 19:402–414. 10.1097/ACI.0000000000000536 31082821 PMC6635048

[42] Valenta R Ferreira F Focke-Tejkl M Linhart B Niederberger V Swoboda I. From allergen genes to allergy vaccines. Annu Rev Immunol. (2010) 28:211–41. 10.1146/annurev-immunol-030409-101218 20192803

[43] Jutel M Jaeger L Suck R Meyer H Fiebig H Cromwell O. Allergen-specific immunotherapy with recombinant grass pollen allergens. J Allergy Clin Immunol. (2005) 116:608–13. 10.1016/j.jaci.2005.06.004 16159631

[44] Pauli G Larsen TH Rak S Horak F Pastorello E Valenta R. Efficacy of recombinant birch pollen vaccine for the treatment of birch-allergic rhinoconjunctivitis. J Allergy Clin Immunol. (2008) 122:951–60. 10.1016/j.jaci.2008.09.017 19000581

[45] Möller C Dreborg S Ferdousi HA Halken S Høst A Jacobsen L. Pollen immunotherapy reduces the development of asthma in children with seasonal rhinoconjunctivitis (the PAT-study). J Allergy Clin Immunol. (2002) 109:251–6. 10.1067/mai.2002.121317 11842293

[46] Holt PG Sly PD Sampson HA Robinson P Loh R Lowenstein H. Prophylactic use of sublingual allergen immunotherapy in high-risk children: A pilot study. J Allergy Clin Immunol. (2013) 132:991–3.e1. 10.1016/j.jaci.2013.04.049 23768574

[47] Szépfalusi Z Bannert C Ronceray L Mayer E Hassler M Wissmann E. Preventive sublingual immunotherapy in preschool children: first evidence for safety and pro-tolerogenic effects. Pediatr Allergy Immunol. (2014) 25:788–95. 10.1111/pai.12310 25406682 PMC6597351

[48] Durham SR Yang WH Pedersen MR Johansen N Rak S. Sublingual immunotherapy with once-daily grass allergen tablets: a randomized controlled trial in seasonal allergic rhinoconjunctivitis. J Allergy Clin Immunol. (2006) 117:802–9. 10.1016/j.jaci.2005.12.1358 16630937

[49] Linhart B Valenta R. Mechanisms underlying allergy vaccination with recombinant hypoallergenic allergen derivatives. Vaccine. (2012) 30:4328–35. 10.1016/j.vaccine.2011.11.011 22100888 PMC4571077

[50] Niederberger V Horak F Vrtala S Spitzauer S Krauth M-T Valent P. Vaccination with genetically engineered allergens prevents progression of allergic disease. Proc Natl Acad Sci USA. (2004) 101:14677–82. 10.1073/pnas.0404735101 15310844 PMC521981

[51] Campana R Moritz K Marth K Neubauer A Huber H Henning R. Frequent occurrence of T cell–mediated late reactions revealed by atopy patch testing with hypoallergenic rBet v 1 fragments. J Allergy Clin Immunol. (2016) 137:601–9.e8. 10.1016/j.jaci.2015.08.042 26518092 PMC4748398

[52] Campana R Marth K Zieglmayer P Weber M Lupinek C Zhernov Y Vaccination of nonallergic individuals with recombinant hypoallergenic fragments of birch pollen allergen Bet v 1: safety, effects, and mechanisms. J Allergy Clin Immunol. (2019) 143:1258–61. 10.1016/j.jaci.2018.11.011 30471304 PMC6411133

[53] Larché M. T cell epitope-based allergy vaccines. Curr Top Microbiol Immunol. (2011) 352:107–19. 10.1007/82_2011_131 21567311

[54] Haselden BM Barry Kay A Larché M. Immunoglobulin E–independent major histocompatibility complex–restricted T cell peptide epitope–induced late asthmatic reactions. J Exp Med. (1999) 189:1885–94. 10.1084/jem.189.12.1885 10377184 PMC2192970

[55] Campbell JD Buckland KF McMillan SJ Kearley J Oldfield WLG Stern LJ. Peptide immunotherapy in allergic asthma generates IL-10–dependent immunological tolerance associated with linked epitope suppression. J Exp Med. (2009) 206:1535–47. 10.1084/jem.20082901 19528258 PMC2715096

[56] Campana R Huang H-J Freidl R Linhart B Vrtala S Wekerle T. Recombinant allergen and peptide-based approaches for allergy prevention by oral tolerance. Semin Immunol. (2017) 30:67–80. 10.1016/j.smim.2017.08.017 28939389

[57] Schmitz N Dietmeier K Bauer M Maudrich M Utzinger S Muntwiler S. Displaying Fel d1 on virus-like particles prevents reactogenicity despite greatly enhanced immunogenicity: a novel therapy for cat allergy. J Exp Med. (2009) 206:1941–55. 10.1084/jem.20090199 19667059 PMC2737174

[58] Storni F Zeltins A Balke I Heath MD Kramer MF Skinner MA. Vaccine against peanut allergy based on engineered virus-Like-Particles displaying single major peanut allergens. J Allergy Clin Immunol. (2019) 145:1240–53.e3. 10.1016/j.jaci.2019.12.007 31866435

[59] Engeroff P Caviezel F Storni F Thoms F Vogel M Bachmann MF. Allergens displayed on virus-like particles are highly immunogenic but fail to activate human mast cells. Allergy. (2018) 73:341–9. 10.1111/all.13268 28787769

[60] Soongrung T Mongkorntanyatip K Peepim T Jitthamstaporn S Pitakpolrat P Kaewamatawong T. Virus-like particles displaying major HDM allergen Der p 2 for prophylactic allergen immunotherapy. Allergy. (2020) 75:1232–6. 10.1111/all.14096 31701528

[61] Kündig TM Senti G Schnetzler G Wolf C Prinz Vavricka BM Fulurija A. Der p 1 peptide on virus-like particles is safe and highly immunogenic in healthy adults. J Allergy Clin Immunol. (2006) 117:1470–6. 10.1016/j.jaci.2006.01.040 16751015

[62] Kueng HJ Manta C Haiderer D Leb VM Schmetterer KG Neunkirchner A. Fluorosomes: a convenient new reagent to detect and block multivalent and complex receptor-ligand interactions. FASEB J. (2010) 24:1572–82. 10.1096/fj.09-137281 20056716 PMC2879947

[63] Derdak SV Kueng HJ Leb VM Neunkirchner A Schmetterer KG Bielek E. Direct stimulation of T lymphocytes by immunosomes: virus-like particles decorated with T cell receptor/CD3 ligands plus costimulatory molecules. Proc Natl Acad Sci USA. (2006) 103:13144–9. 10.1073/pnas.0602283103 16924110 PMC1559767

[64] Kratzer B Hofer S Zabel M Pickl WF. All the small things: how virus-like particles and liposomes modulate allergic immune responses. Eur J Immunol. (2020) 50:17–32. 10.1002/eji.201847810 31799700 PMC6973265

[65] Neunkirchner A Kratzer B Köhler C Smole U Mager LF Schmetterer KG. Genetic restriction of antigen-presentation dictates allergic sensitization and disease in humanized mice. EBioMedicine. (2018) 31:66–78. 10.1016/j.ebiom.2018.04.001 29678672 PMC6014064

[66] Kratzer B Köhler C Hofer S Smole U Trapin D Iturri J. Prevention of allergy by virus-like nanoparticles (VNP) delivering shielded versions of major allergens in a humanized murine allergy model. Allergy. (2019) 74:246–60. 10.1111/all.13573 30035810 PMC6587790

[67] Valenta R Campana R Focke-Tejkl M Niederberger V. Vaccine development for allergen-specific immunotherapy based on recombinant allergens and synthetic allergen peptides: Lessons from the past and novel mechanisms of action for the future. J Allergy Clin Immunol. (2016) 137:351–7. 10.1016/j.jaci.2015.12.1299 26853127 PMC4861208

[68] Valenta R Campana R Niederberger V. Recombinant allergy vaccines based on allergen-derived B cell epitopes. Immunol Lett. (2017) 189:19–26. 10.1016/j.imlet.2017.04.015 28472641 PMC6390931

[69] Edlmayr J Niespodziana K Linhart B Focke-Tejkl M Westritschnig K Scheiblhofer S A combination vaccine for allergy and rhinovirus infections based on rhinovirus-derived surface protein VP1 and a nonallergenic peptide of the major timothy grass pollen allergen Phl p 1. J Immunol. (2009) 182:6298–306. 10.4049/jimmunol.0713622 19414783

[70] Edlmayr J Niespodziana K Focke-Tejkl M Linhart B Valenta R. Allergen-specific immunotherapy: towards combination vaccines for allergic and infectious diseases, Curr Topics Microbiol Immunol. (2011) 352:121–140. 10.1007/82_2011_130 21626347

[71] Banerjee S Weber M Blatt K Swoboda I Focke-Tejkl M Valent P. Conversion of der p 23, a new major house dust mite allergen, into a hypoallergenic vaccine. J Immunol. (2014) 192:4867–75. 10.4049/jimmunol.1400064 24733847 PMC4582415

[72] Marth K Breyer I Focke-Tejkl M Blatt K Shamji MH Layhadi J. A Nonallergenic birch pollen allergy vaccine consisting of hepatitis PreS–Fused Bet v 1 peptides focuses blocking igg toward ige epitopes and shifts immune responses to a tolerogenic and Th1 phenotype. J Immunol. (2013) 190:3068–78. 10.4049/jimmunol.1202441 23440415 PMC4148560

[73] Focke-Tejkl M Weber M Niespodziana K Neubauer A Huber H Henning R. Development and characterization of a recombinant, hypoallergenic, peptide-based vaccine for grass pollen allergy. J Allergy Clin Immunol. (2015) 135:1207–17.e11. 10.1016/j.jaci.2014.09.012 25441634 PMC4418753

[74] Niespodziana K Focke-Tejkl M Linhart B Civaj V Blatt K Valent P. A hypoallergenic cat vaccine based on Fel d 1–derived peptides fused to hepatitis B PreS. J Allergy Clin Immunol. (2011) 127:1562–70.e6. 10.1016/j.jaci.2011.02.004 21411130 PMC6624143

[75] Weber M Niespodziana K Linhart B Neubauer A Huber H Henning R. Comparison of the immunogenicity of BM32, a recombinant hypoallergenic B cell epitope–based grass pollen allergy vaccine with allergen extract–based vaccines. J Allergy Clin Immunol. (2017) 140:1433–6.e6. 10.1016/j.jaci.2017.03.048 28576673 PMC6392172

[76] Niederberger V Marth K Eckl-Dorna J Focke-Tejkl M Weber M Hemmer W. Skin test evaluation of a novel peptide carrier–based vaccine, BM32, in grass pollen–allergic patients. J Allergy Clin Immunol. (2015) 136:1101–3.e8. 10.1016/j.jaci.2015.03.034 26048664 PMC6536382

[77] Zieglmayer P Focke-Tejkl M Schmutz R Lemell P Zieglmayer R Weber M. Mechanisms, safety and efficacy of a B cell epitope-based vaccine for immunotherapy of grass pollen allergy. EBioMedicine. (2016) 11:43–57. 10.1016/j.ebiom.2016.08.022 27650868 PMC5049999

[78] Niederberger V Neubauer A Gevaert P Zidarn M Worm M Aberer W. Safety and efficacy of immunotherapy with the recombinant B-cell epitope–based grass pollen vaccine BM32. J Allergy Clin Immunol. (2018) 142:497–509.e9. 10.1016/j.jaci.2017.09.052 29361332 PMC6392176

[79] Rauber MM Möbs C Campana R Henning R Schulze-Dasbeck M Greene B. Allergen immunotherapy with the hypoallergenic B cell epitope-based vaccine BM32 modifies IL10 and IL5-secreting T cells. Allergy. (2019) 75:450–3. 10.1111/all.13996 31330050

[80] Eckl-Dorna J Weber M Stanek V Linhart B Ristl R Waltl EE. Two years of treatment with the recombinant grass pollen allergy vaccine BM32 induces a continuously increasing allergen-specific IgG4 response. EBioMedicine. (2019) 50:421–32. 10.1016/j.ebiom.2019.11.006 31786130 PMC6921329

[81] Cornelius C Schöneweis K Georgi F Weber M Niederberger V Zieglmayer P. Immunotherapy with the PreS-based grass pollen allergy vaccine BM32 induces antibody responses protecting against hepatitis B infection. EBioMedicine. (2016) 11:58–67. 10.1016/j.ebiom.2016.07.023 27568223 PMC5049759

[82] Matricardi PM Kleine-Tebbe J Hoffmann HJ Valenta R Hilger C Hofmaier S. EAACI molecular allergology user's guide. Pediatr Allergy Immunol. (2016) 27:1–250. 10.1111/pai.12563 27288833

[83] Elisyutina O Fedenko E Campana R Litovkina A Ilina N Kudlay D. Bet v 1specific IgE levels and PR10 reactivity discriminate silent sensitization from phenotypes of birch allergy. Allergy. (2019) 74:2525–8. 10.1111/all.13931 31145475 PMC6911368

[84] Thomas WR. Geography of house dust mite allergens. Asian Pacific J Allergy Immunol. (2010) 28:211–24. 21337903

[85] Chen Z-G Li Y-T Wang W-H Tan K Sen Zheng R Yang L-F. Distribution and determinants of *dermatophagoides* mites sensitization ofallergic rhinitis and allergic asthma in china. Int Arch Allergy Immunol. (2019) 180:17–27. 10.1159/000499409 31104060

[86] Huang H-J Resch-Marat Y Rodriguez-Dominguez A Chen K-W Kiss R Zieglmayer P. Underestimation of house dust mite–specific IgE with extract-based ImmunoCAPs compared with molecular immunoCAPs. J Allergy Clin Immunol. (2018) 142:1656–59.e9. 10.1016/j.jaci.2018.07.010 30059698

[87] Huang H Curin M Banerjee S Chen K Garmatiuk T Resch-Marat Y. A hypoallergenic peptide mix containing T cell epitopes of the clinically relevant house dust mite allergens. Allergy. (2019) 74:2461–78. 10.1111/all.13956 31228873 PMC7078969

[88] Gangl K Niederberger V Valenta R. Multiple grass mixes as opposed to single grasses for allergen immunotherapy in allergic rhinitis. Clin Exp Allergy. (2013) 43:1202–16. 10.1111/cea.12128 24152153 PMC6624134

[89] Villalba M Rodríguez R Batanero E. The spectrum of olive pollen allergens. From structures to diagnosis and treatment. Methods. (2014) 66:44–54. 10.1016/j.ymeth.2013.07.038 23920474

[90] Dorofeeva Y Colombo P Blanca M Mari A Khanferyan R Valenta R. Expression and characterization of recombinant Par j 1 and Par j 2 resembling the allergenic epitopes of *Parietaria judaica* pollen. Sci Rep. (2019) 9:15043. 10.1038/s41598-019-50854-1 31636285 PMC6803649

[91] Wang XY Ma TT Wang XY Zhuang Y Wang XD Ning HY. Prevalence of pollen-induced allergic rhinitis with high pollen exposure in grasslands of Northern China. Allergy. (2018) 73:1232–43. 10.1111/all.13388 29322523 PMC6033040

[92] Ma T Wang X Zhuang Y Shi H Ning H Lan T. Prevalence and risk factors for allergic rhinitis in adults and children living in different grassland regions of Inner Mongolia. Allergy. (2020) 75:234–9. 10.1111/all.13941 31169905

[93] Penagos M Durham SR. Duration of allergen immunotherapy for inhalant allergy. Curr Opin Allergy Clin Immunol. (2019) 19:594–605. 10.1097/ACI.0000000000000585 31464717

[94] Jarrett E Hall E. Selective suppression of IgE antibody responsiveness by maternal influence. Nature. (1979) 280:145–7. 10.1038/280145a0 95350

[95] Jarrett EEE Hall E. IgE suppression by maternal IgG. Immunology. (1983) 48:49–58. 6848454 PMC1454004

[96] Victor JR Fusaro AE Duarte AJ da S Sato MN. Preconception maternal immunization to dust mite inhibits the type I hypersensitivity response of offspring. J Allergy Clin Immunol. (2003) 111:269–77. 10.1067/mai.2003.39 12589344

[97] Uthoff H Spenner A Reckelkamm W Ahrens B Wölk G Hackler R. Critical role of preconceptional immunization for protective and nonpathological specific immunity in murine neonates. J Immunol. (2003) 171:3485–92. 10.4049/jimmunol.171.7.3485 14500644

[98] Burton OT Tamayo JM Stranks AJ Koleoglou KJ Oettgen HC. Allergen-specific IgG antibody signaling through FcγRIIb promotes food tolerance. J Allergy Clin Immunol. (2018) 141:189–201.e3. 10.1016/j.jaci.2017.03.045 28479335 PMC5671359

[99] Freidl R Gstoettner A Baranyi U Swoboda I Stolz F Focke-Tejkl M. Blocking antibodies induced by immunization with a hypoallergenic parvalbumin mutant reduce allergic symptoms in a mouse model of fish allergy. J Allergy Clin Immunol. (2017) 139:1897–905.e1. 10.1016/j.jaci.2016.10.018 27876628 PMC5438872

[100] Linhart B Narayanan M Focke-Tejkl M Wrba F Vrtala S Valenta R Prophylactic and therapeutic vaccination with carrier-bound Bet v 1 peptides lacking allergen-specific T cell epitopes reduces Bet v 1-specific T cell responses via blocking antibodies in a murine model for birch pollen allergy. Clin Exp Allergy. (2014) 44:278–87. 10.1111/cea.12216 24447086 PMC4215111

[101] Flicker S Linhart B Wild C Wiedermann U Valenta R. Passive immunization with allergen-specific IgG antibodies for treatment and prevention of allergy. Immunobiology. (2013) 218:884–91. 10.1016/j.imbio.2012.10.008 23182706 PMC3636530

[102] Glovsky MM Ghekiere L Rejzek E. Effect of maternal immunotherapy on immediate skin test reactivity, specific rye I IgG and IgE antibody, and total IgE of the children. Ann Allergy. (1991) 67:21–4. 1859036

[103] Flicker S Marth K Kofler H Valenta R. Placental transfer of allergen-specific IgG but not IgE from a specific immunotherapy–treated mother. J Allergy Clin Immunol. (2009) 124:1358–360.e1. 10.1016/j.jaci.2009.09.024 20004788

[104] Buchy P Badur S Kassianos G Preiss S Tam JS. Vaccinating pregnant women against influenza needs to be a priority for all countries: an expert commentary. Int J Infect Dis. (2020) 92:1–12. 10.1016/j.ijid.2019.12.019 31863875

[105] Jarvis JR Dorey RB Warricker FDM Alwan NA Jones CE. The effectiveness of influenza vaccination in pregnancy in relation to child health outcomes: systematic review and meta-analysis. Vaccine. (2020) 38:1601–13. 10.1016/j.vaccine.2019.12.056 31932138

[106] Seale AC Baker CJ Berkley JA Madhi SA Ordi J Saha SK. Vaccines for maternal immunization against group B Streptococcus disease: WHO perspectives on case ascertainment and case definitions. Vaccine. (2019) 37:4877–85. 10.1016/j.vaccine.2019.07.012 31303524 PMC6677922

[107] D'Heilly C Switzer C Macina D. Safety of maternal immunization against pertussis: a systematic review. Infect Dis Ther. (2019) 8:543–68. 10.1007/s40121-019-00265-6 31531826 PMC6856234

[108] Psarris A Sindos M Daskalakis G Chondrogianni ME Panayiotou S Antsaklis P. Immunizations during pregnancy: how, when and why. Eur J Obstet Gynecol Reprod Biol. (2019) 240:29–35. 10.1016/j.ejogrb.2019.06.019 31226574

[109] Kochhar S Edwards KM Ropero Alvarez AM Moro PL Ortiz JR. Introduction of new vaccines for immunization in pregnancy – programmatic, regulatory, safety and ethical considerations. Vaccine. (2019) 37:3267–77. 10.1016/j.vaccine.2019.04.075 31072733 PMC6771279

[110] Maertens K Orije MRP Van Damme P Leuridan E. Vaccination during pregnancy: current and possible future recommendations. Eur J Pediatr. (2020) 179:235–42. 10.1007/s00431-019-03563-w 31912233 PMC7222942

